# Chronic kidney disease in nonalcoholic fatty liver disease at primary healthcare centers in Korea

**DOI:** 10.1371/journal.pone.0279367

**Published:** 2022-12-20

**Authors:** Eun-Hee Nah, Sug Kyun Shin, Seon Cho, Hyeran Park, Suyoung Kim, Eunjoo Kwon, Han-Ik Cho

**Affiliations:** 1 Health Promotion Research Institute, Korea Association of Health Promotion, Seoul, Korea; 2 Department of Internal Medicine, National Health Insurance Service Ilsan Hospital, Goyang, Korea; 3 MEDIcheck LAB, Korea Association of Health Promotion, Seoul, Korea; Bolu Abant İzzet Baysal University: Bolu Abant Izzet Baysal Universitesi, TURKEY

## Abstract

**Background:**

The prevalence rates of nonalcoholic fatty liver disease (NAFLD) and chronic kidney disease (CKD) are expected to increase with the rising trends in diabetes and obesity associated with aging populations. Considering the impacts of coexistent NAFLD and CKD on morbidity and mortality rates, screening strategies for groups at high-risk of CKD are needed in community-dwelling individuals with NAFLD. The aims of this study were to determine the prevalence and distribution of CKD in NAFLD, as well as the risk factors for CKD and the correlation with liver fibrosis in asymptomatic individuals with NAFLD at primary healthcare centers in Korea.

**Methods:**

This retrospective cross-sectional study used data from 13 health-promotion centers in 10 Korean cities. Liver steatosis and stiffness were assessed using ultrasonography and magnetic resonance elastography (MRE), respectively. CKD was defined as an estimated glomerular filtration rate of <60 mL/min/1.73m^2^, and urine albumin-to-creatinine ratio or proteinuria. CKD was categorized into four stages: no CKD, mild, moderate, and severe. Comparisons according to the CKD stages in NAFLD were performed using Student’s *t*-test or the chi-square test. Multivariable logistic regression analyses were performed to identify the risk factors for CKD and the correlation with liver fibrosis in NAFLD.

**Results:**

The prevalence of CKD was 12.4% in NAFLD. Albuminuria (16.2%) and proteinuria (8.0%) were more prevalent in NAFLD. NAFLD (odd ratio = 1.27, 95% CI = 1.09–1.48, *P* = 0.003) was independently associated with CKD of at least mild stage. However, there was no significant association between CKD of at least moderate stage and NAFLD after adjusting for age and a metabolically unhealthy status. CKD was associated with significant liver fibrosis as measured by MRE in NAFLD.

**Conclusion:**

The presence of NAFLD and liver fibrosis were independent risk factors for CKD, but NAFLD was not an independent risk factor for the later stages of CKD.

## Introduction

Nonalcoholic fatty liver disease (NAFLD) is a growing global health concern whose reported prevalence has ranged from 8% to 45% among the general population [1]. Its increasing prevalence is expected to continue with the rising trends in obesity and diabetes in aging societies. Although cirrhosis and its complications are the most common liver-related causes of morbidity, cardiovascular diseases (CVDs) are the leading cause of overall morbidity and mortality in NAFLD [2]. In addition, some studies have shown that NAFLD is the underlying cause not only for a wide spectrum of liver damage, but also for several extrahepatic manifestations including chronic kidney diseases (CKD) [3,4].

CKD is expected to be one of the leading causes of death globally in the near future [5]. The prevalence of CKD has been estimated at 9–15% [6,7]. Some studies found that the prevalence and risk of CKD were significantly increased among patients with NAFLD and that CKD was independently associated with an increased overall mortality in NAFLD [8–10]. However, there are also controversies on the relationship between NAFLD and CKD according to regions, races, and other characteristics of study populations [11–13]. NAFLD and CKD share some cardiometabolic risk factors that lead to CVD events in both diseases [14,15]. Furthermore, more advanced NAFLD has a greater impact on incident CKD. Considering the impacts of coexistent NAFLD and CKD on morbidity and mortality rates, screening strategies for groups at high-risk of CKD are needed in community-dwelling individuals with NAFLD.

The aims of this study were to determine the prevalence and distribution of CKD in NAFLD, as well as the risk factors for CKD and the correlation with liver fibrosis in asymptomatic individuals with NAFLD at primary healthcare centers in Korea.

## Materials and methods

### Study subjects

This cross-sectional, retrospective study consecutively selected subjects who underwent health checkups including magnetic resonance elastography (MRE), abdominal ultrasonography (US), and renal function tests at 13 health-promotion centers in 10 Korean cities between 2018 and 2021. The Korea Association of Health Promotion is running a health checkup program that includes those provided by the Korean National Health Insurance Service (NHIS) but also programs that are paid for privately. This program involves 17 health-promotion centers in 13 cities, and the 13 of these health-promotion centers that have MRE facilities were selected for inclusion in the current study. The medical records and lifestyle information of the subjects were also reviewed. The exclusion criteria were a history of viral hepatitis or hepatocellular malignancy, secondary causes of fatty liver or high alcohol consumption (>210 g for males and > 140 g for females weekly). Analyses were applied to 8,909 eligible subjects. The study protocol was reviewed and approved by the institutional review board of the Korea Association of Health Promotion (approval no.: 130750-202206-HR-002). The requirement for informed consent was waived due to the retrospective design of the study, and the analyses were performed on anonymous clinical data.

### Fatty liver assessment

The presence and degree of fatty liver were evaluated by US. The parenchymal brightness, liver-to-kidney contrast, deep beam attenuation, and bright vessel walls were used as standard criteria for diagnosing fatty liver [16].

### Liver fibrosis measurements

MRE was performed using either MRE hardware (GE Healthcare, Waukesha, WI, USA) with a 1.5-T imaging system or a 1.5-T whole-body magnetic resonance unit (Gyroscan Intera, Philips Medical Systems, Best, the Netherlands) with a four-element torso coil. The two-dimensional MRE protocols used were similar to those described in the literature [17,18]. Liver stiffness (LS) values were calculated as the median values in multiple regions of interest on elastograms. The cutoff values for significant and advanced hepatic fibrosis were based on the MRE standards for LS of 2.91–3.59 kPa and ≥3.60 kPa, respectively [19,20]. The NAFLD fibrosis score (NFS) was calculated using the following formula: –1.675 + 0.037 × age (years) + 0.094 × BMI (kg/m^2^) + 1.13 × impaired fasting glucose/diabetes (yes = 1, no = 0) + 0.99 × AST (aspartate aminotransferase)/ALT (alanine aminotransferase) ratio– 0.013 × platelet count (× 10^9^/L)– 0.66 × albumin (g/dL). The Fibrosis-4 Index (FIB-4) was calculated using the following formula: age × AST (IU/L) / platelet count (10^9^/L) × √ALT (IU/L) [21].

### Laboratory measurements and assessment of CKD

Each blood sample was collected from the antecubital vein of each subject in a sitting position after fasting for >8 hours, and random spot urine samples were also obtained from the subjects. The biochemical measurements such as blood glucose, lipids, and serum creatinine, were made using the Hitachi 7600 analyzer (Hitach, Tokyo, Japan). Metabolic syndrome (MS) was defined according to the National Cholesterol Education Program ATP III criteria [22]. A metabolically unhealthy status was defined as having two or more components of MS and/or diabetes.

Albumin-to-creatinine ratio (ACR) and proteinuria were measured using a urine test strip analyzer UC-3500 (Sysmex, Kobe, Japan). Test strips (Meditape UC-11A, Sysmex, Kobe, Japan) were used in this study. A semiquantitative ACR of ≥30 mg/g is considered to indicate albuminuria. Urinary protein was detected based on the protein error of a pH indicator, with proteinuria reported as trace, 1+, 2+, or 3+, which corresponds to a protein level of 15, 30, 100, or 300 mg/dL, respectively. The serum creatinine concentration was measured using the Jaffe rate-blanked colorimetric method with the Hitachi Automatic Analyzer 7600 (Hitachi, Tokyo, Japan). The estimated glomerular filtration rate (eGFR) was calculated using the following equation from the Modification of Diet in Renal Disease study (MDRD): eGFR (mL/min/1.73 m^2^) = 175 × [serum creatinine (mg/dL)]^–1.154^ × [age (years)]^–0.203^ × (0.742 if female) [23]. CKD was defined as eGFR < 60 mL/min/1.73 m^2^ and/or ACR ≥30 mg/g and/or proteinuria ≥+1. In accordance with the Kidney Diseases Improving Global Outcomes (KDIGO) staging system, we categorized eGFR values into G1, G2, G3a, G3b, and G4/5, corresponding to eGFR ≥90, 60–89, 45–59, 30–44, and <30 mL/min/1.73 m^2^, respectively. Albuminuria was categorized into A1, A2, and A3, corresponding to ACR <30, 30–300, and >300 mg/g, respectively. The severity of CKD was categorized into four stages based on the National Institute of Diabetes and KDIGO: no CKD (G1/2-A1), mild CKD (G3a-A1 or G1/2-A2), moderate CKD (G3b-A1, G3a-A2, or G1/2-A3), and severe CKD (G4/5-A1, G3b–5-A2, or G3a–5-A3) [24].

### Statistical analysis

Statistical analyses were performed using SAS version 9.4 (SAS Institute, Cary, NC, USA). Data are presented as mean ± standard deviation and frequency (percentage) values. The differences in the subject’s characteristics were analyzed according to the presence of NAFLD using Student’s *t*-test or the chi-square test. Differences between the four CKD groups were analyzed using one-way ANOVAs and chi-square tests. Univariate and multivariable logistic regression analyses were performed to identify the associations of CKD with metabolic abnormalities and NAFLD, and to evaluate the association of liver fibrosis with CKD. *P* values of <0.05 were considered significant.

## Results

**Fig 1:**
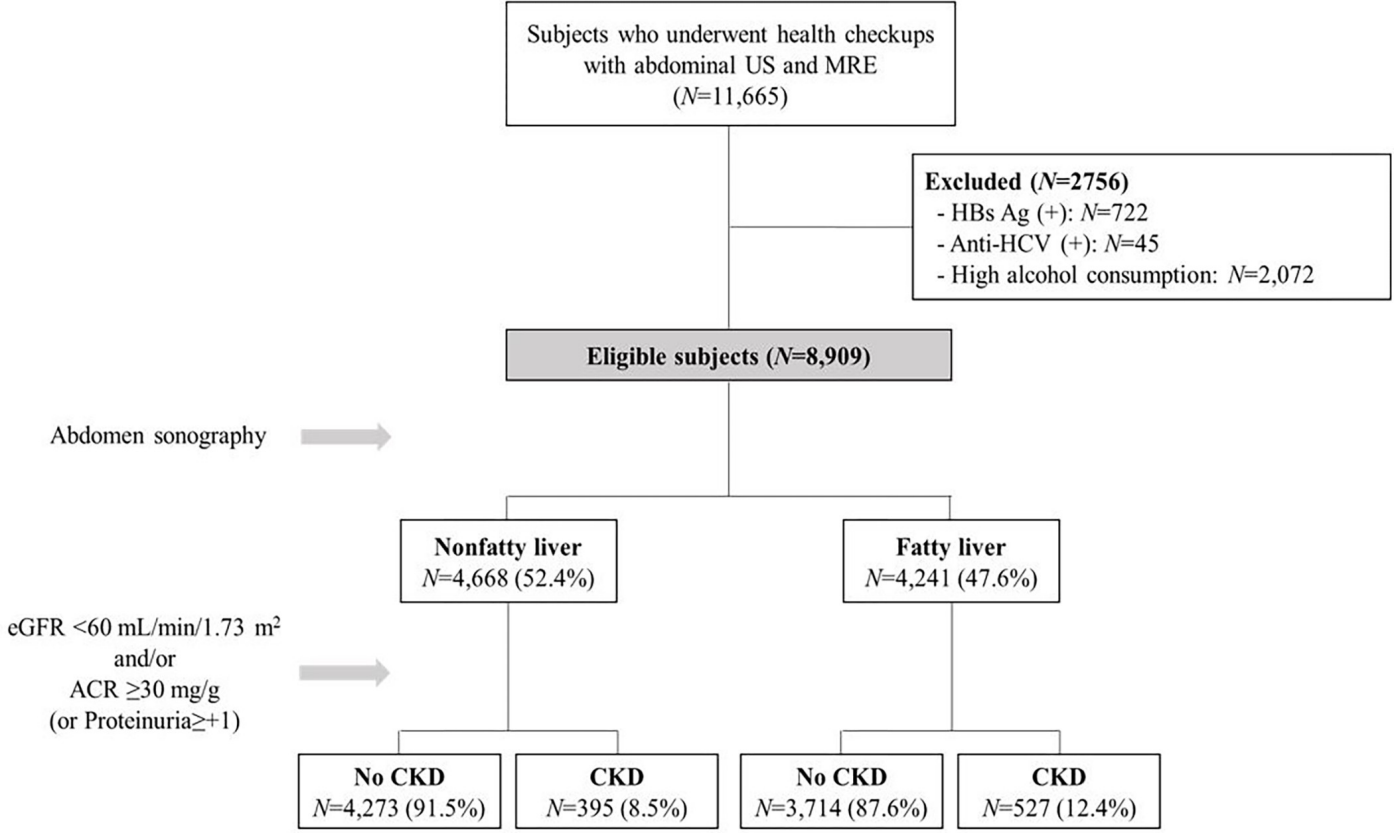
Flow chart of the study.

The 11,665 initially enrolled subjects who underwent health checkup including MRE, abdominal US and renal function tests were consecutively selected from 13 health-promotion centers in Korea. After applying the exclusion criteria, 8,909 subjects were finally included in the study. Fatty liver was detected in 4,241 (47.6%) subjects by abdominal US (Fig 1).

### Characteristics of the study subject according to the presence of NAFLD

The subjects were aged 49.2±11.0 years and their eGFR was 79.5±12.8 mL/min/1.73 m^2^. CKD was more prevalent in the NAFLD than the non-NAFLD group (12.4% vs.8.5%). The eGFR was significantly lower in the NAFLD group (80.1±12.9 vs. 78.9±12.6 mL/min/1.73 m^2^), while the prevalence of eGFR < 60 mL/min/1.73 m^2^ did not differ significantly between the NAFLD and non-NAFLD groups. The prevalence rates of albuminuria and proteinuria were higher in the NAFLD than the non-NAFLD group (16.2% vs. 8.2%, and 8.0% vs. 4.7%, respectively). Compared with subjects without NAFLD, those with NAFLD were more likely to be older, male, have a larger waist circumference, and have hypertension, prediabetes, and MS. The LS as measured by MRE was also higher in the NAFLD group (Table 1).

**Table 1:** Characteristics of study subjects with or without NAFLD.

Variables		Total		Non-NAFLD		NAFLD		P-value
		(N = 8,909)		(N = 4,668)		(N = 4,241)		
Age, years		49.2	(± 11.0)	48.8	(± 11.7)	49.7	(± 10.2)	<0.001
Sex, male		7007	(78.7%)	3266	(70%)	3741	(88.2%)	<0.001
Serum creatinine, mg/dL		1.0	(± 0.2)	1.0	(± 0.3)	1.1	(± 0.2)	<0.001
eGFR, mL/min/1.73m^2^		79.5	(± 12.8)	80.1	(± 12.9)	78.9	(± 12.6)	<0.001
eGFR, <60mL/min/1.73m²		348	(3.9%)	172	(3.7%)	176	(4.2%)	0.258
ACR, ≥30mg/g		161	(11.8%)	61	(8.2%)	100	(16.2%)	<0.001
Proteinuria, ≥+1		557	(6.3%)	219	(4.7%)	338	(8.0%)	<0.001
eGFR categories								
	G1	1784	(20%)	1006	(21.6%)	778	(18.3%)	0.011
	G2	6777	(76.1%)	3490	(74.8%)	3287	(77.5%)	
	G3a	323	(3.6%)	160	(3.4%)	163	(3.8%)	
	G3b	21	(0.2%)	10	(0.2%)	11	(0.3%)	
	G4	2	(0.02%)	1	(0.02%)	1	(0.02%)	
	G5	2	(0.02%)	1	(0.02%)	1	(0.02%)	
CKD categories								
	No CKD	7987	(89.7%)	4273	(91.5%)	3714	(87.6%)	<0.001
	Mild	772	(8.7%)	336	(7.2%)	436	(10.3%)	
	Moderate	114	(1.3%)	42	(0.9%)	72	(1.7%)	
	Severe	36	(0.4%)	17	(0.4%)	19	(0.5%)	
CKD (≥Mild)		922	(10.4%)	395	(8.5%)	527	(12.4%)	<0.001
Waist circumference, cm		85.3	(± 9.5)	80.7	(± 8.2)	90.3	(± 8.1)	<0.001
Hypertension		2625	(29.5%)	978	(21%)	1647	(38.9%)	<0.001
Pre-diabetes		4595	(51.6%)	1916	(41.1%)	2679	(63.2%)	<0.001
FBS, mg/g		99.6	(± 21.1)	95.3	(± 16.5)	104.3	(± 24.4)	<0.001
HbA1c, %		5.8	(± 0.8)	5.6	(± 0.7)	6.0	(± 0.9)	<0.001
TG, mg/dL		138.1	(± 103.3)	106.3	(± 73.7)	173	(± 118.8)	<0.001
HDL-C, mg/dL		52.5	(± 13.0)	56.7	(± 13.6)	47.8	(± 10.6)	<0.001
AST, IU/L		30.2	(± 17.4)	26.8	(± 14.1)	34.0	(± 19.8)	<0.001
ALT, IU/L		33.0	(± 29.0)	24.1	(± 18.7)	42.7	(± 34.6)	<0.001
Metabolic syndrome		2015	(22.8%)	373	(8%)	1642	(39%)	<0.001
MRE, kPa		2.3	(± 0.5)	2.2	(± 0.5)	2.3	(± 0.5)	<0.001
NFS		-2.1	(± 1.3)	-2.2	(± 1.3)	-2.0	(± 1.2)	<0.001
FIB-4		1.2	(± 0.7)	1.2	(± 0.7)	1.1	(± 0.7)	<0.001

Data are mean±standard deviation or N (%) values.

**Abbreviations** NAFLD, nonalcoholic fatty liver disease; eGFR, estimated glomerular filtration rate; ACR, albumin creatinine ratio; CKD, chronic kidney disease; FBS, fasting blood sugar; HbA1c, hemoglobin A1C; TG, triglyceride; HDL-C, high density lipoprotein cholesterol; AST, aspartate aminotransferase; ALT, alanine aminotransferase; MRE, magnetic resonance elastography; NFS, NAFLD fibrosis score; FIB-4, fibrosis-4.

### Associations of CKD with metabolic abnormalities and NAFLD

In the univariate model, age, central obesity, hypertension, diabetes, prediabetes, dyslipidemia, raised liver transaminases, metabolically unhealthy status, MS, and NAFLD were associated with CKD of at least mild stage (all *P*<0.001). In multivariable analysis, after adjusting for age and a metabolically unhealthy status, NAFLD (odds ratio [OR] = 1.27, 95% confidence interval [CI] = 1.09–1.48, *P* = 0.003) was still associated with CKD of at least mild stage. However, age (OR = 1.03, 95% CI = 1.01–1.04) and a metabolically unhealthy status (OR = 3.3, 95% CI = 2.19–4.97) were also significantly associated with CKD of at least moderate stage (all *P*<0.01), while there was no significant association between CKD of at least moderate stage and NAFLD (Table 2).

**Table 2:** Association of CKD with metabolic abnormalities and NAFLD in study subjects.

Variables		CKD					
		≥ Mild			≥ Moderate		
		OR	95% CI	P-value	OR	95% CI	P-value
**Univariate analysis**							
Age, years		1.03	1.03–1.04	<0.001	1.03	1.02–1.0	<0.001
Sex, male (ref: female)		0.97	0.82–1.15	0.722	1.36	0.89–2.10	0.160
Metabolic abnormalities							
	Central obesity	1.72	1.49–1.97	<0.001	3.1	2.23–4.30	<0.001
	Hypertension	2.12	1.85–2.44	<0.001	3.28	2.37–4.55	<0.001
	Diabetes	3.18	2.68–3.76	<0.001	5.53	3.95–7.74	<0.001
	Pre-diabetes	1.80	1.57–2.06	<0.001	2.4	1.74–3.33	<0.001
	TG, ≥150mg/dL	1.40	1.21–1.61	<0.001	1.81	1.31–2.52	<0.001
	Decreased HDL-C	1.20	1.01–1.42	0.038	1.85	1.29–2.66	0.001
	Raised liver transaminases ^†^	1.70	1.48–1.95	<0.001	1.59	1.15–2.20	0.006
	Two or more components of MS	2.03	1.77–2.34	<0.001	3.92	2.69–5.73	<0.001
	Metabolic syndrome	2.05	1.77–2.37	<0.001	3.53	2.54–4.89	<0.001
NAFLD		1.54	1.34–1.76	<0.001	1.71	1.23–2.38	0.001
**Multivariate analysis**							
Age, years		1.03	1.02–1.04	<0.001	1.03	1.01–1.04	0.001
Sex, male (ref: female)		0.88	0.74–1.05	0.155	1.17	0.75–1.82	0.503
Metabolically unhealthy		1.67	1.43–1.96	<0.001	3.3	2.19–4.97	<0.001
NAFLD		1.27	1.09–1.48	0.003	1.08	0.75–1.54	0.693

^†^ Raised liver transaminases: AST>33 IU/L and/or ALT > 38 IU/L.

**Abbreviations** OR, odds ratio; CI, confidence interval.

### Metabolic abnormalities and liver fibrosis according to CKD stages in NAFLD

Those with mild or moderate CKD were more likely to be older and have central obesity, hypertension, prediabetes, hypertriglyceridemia, raised liver transaminases, and MS than were subjects without CKD (all *P*<0.001). In addition, higher proportions of subjects in the mild- and moderate-CKD groups had significant (F2) or advanced fibrosis (F3) (*P*<0.001). The liver fibrosis scores (i.e., LS, NFS, and FIB-4, as estimations of hepatic fibrosis) were also higher in those with mild or moderate CKD than in those without CKD (all *P*<0.001) (Table 3).

**Table 3:** Comparison of metabolic abnormalities and liver fibrosis according to CKD stages.

Variables		Overall NAFLD (N = 4,241)		CKD stages in NAFLD								P-value
				no CKD (N = 3,714)		Mild (N = 436)		Moderate (N = 72)		Severe (N = 19)		
Age, years, Mean±SD		49.7	(±10.2)	49.3	(±10.1)^a^	52.8	(±10.9)^b^	51.3	(±9.9)^a^^,^^b^	58	(±10.4)^b^	<0.001
Sex, male		3741	(88.2%)	3291	(88.6%)	373	(85.6%)	62	(86.1%)	15	(79.0%)	0.144
Metabolic abnormality												
	Central obesity	2225	(52.7%)	1905	(51.5%)	249	(57.2%)	56	(77.8%)	15	(79.0%)	<0.001
	Hypertension	1647	(38.9%)	1380	(37.2%)	210	(48.2%)	46	(63.9%)	11	(57.9%)	<0.001
	Pre-diabetes	1996	(47.1%)	1677	(45.2%)	255	(58.6%)	52	(72.2%)	12	(63.2%)	<0.001
	TG, ≥150mg/dL	2035	(48.4%)	1759	(47.7%)	220	(51%)	48	(67.6%)	8	(42.1%)	0.005
	Decreased HDL-C	1074	(25.5%)	936	(25.4%)	107	(24.8%)	24	(33.8%)	7	(36.8%)	0.263
	Raised liver transaminases ^†^	1978	(46.6%)	1681	(45.3%)	250	(57.3%)	40	(55.6%)	7	(36.8%)	<0.001
	Metabolic syndrome	1634	(39.0%)	1365	(37.2%)	205	(47.7%)	53	(74.7%)	11	(57.9%)	<0.001
MRE category*												
	F0	923	(21.9%)	809	(21.9%)	100	(23.1%)	11	(15.5%)	3	(16.7%)	<0.001
	F1	2978	(70.6%)	2634	(71.2%)	286	(66.1%)	48	(67.6%)	10	(55.6%)	
	F2	251	(5.9%)	207	(5.6%)	30	(6.9%)	9	(12.7%)	5	(27.8%)	
	F3	69	(1.6%)	49	(1.3%)	17	(3.9%)	3	(4.2%)	-	(0.0%)	
	MRE, kPa	2.31	(±0.54)	2.29	(±0.52)^a^	2.37	(±0.63)^a^^,^^b^	2.52	(±0.71)^b^	2.48	(±0.55)^a^^,^^b^	<0.001
NFS category												
	< -1.455	2594	(67.1%)	2319	(68.3%)	235	(60.3%)	35	(56.5%)	5	(33.3%)	<0.001
	-1.455 ≤ NFS < 0.676	1213	(31.4%)	1034	(30.5%)	147	(37.7%)	24	(38.7%)	8	(53.3%)	
	≥ 0.676	56	(1.4%)	43	(1.3%)	8	(2.1%)	3	(4.8%)	2	(13.3%)	
	NFS value	-2.009	(±1.239)	-2.047	(±1.229)^a^	-1.797	(±1.272)^b^	-1.556	(±1.206)^b^	-0.981	(±1.598)^b^	<0.001
FIB-4 category												
	< 1.0	2091	(49.9%)	1875	(51%)	178	(41.4%)	34	(47.9%)	4	(21.1%)	<0.001
	1.0 ≤ FIB-4 < 1.3	896	(21.4%)	802	(21.8%)	70	(16.3%)	18	(25.4%)	6	(31.6%)	
	≥ 1.3	1207	(28.8%)	997	(27.1%)	182	(42.3%)	19	(26.8%)	9	(47.4%)	
	FIB-4 value	1.1	(±0.7)	1.1	(±0.6)^a^	1.4	(±0.9)^b^	1.2	(±0.7)^a^^,^^b^	1.6	(±0.9)^b^	<0.001

Data are mean±standard deviation or N (%) values.

† Raised liver transaminases: AST>33 IU/L and/or ALT > 38 IU/L.

*The stage of liver fibrosis using MRE are defined as F0 (normal liver stiffness; <1.94), F1 (mild fibrosis; 1.95–2.9), F2 (significant fibrosis; 2.91–3.59) and F3 (advanced fibrosis; 33.6).

^a,b,c,d^: Different letters indicate a significant difference between CKD stages using Scheffe’s multiple-comparisons test.

**Abbreviations** MRE, magnetic resonance elastography; NFS, NAFLD fibrosis score; FIB-4, fibrosis-4.

### Association of CKD with liver fibrosis in NAFLD

In multivariable analysis, after adjusting for age, central obesity, hypertension, prediabetes, and hypertriglyceridemia, advanced liver fibrosis (OR = 2.00, 95% CI = 1.13–3.55, *P* = 0.018) as measured by MRE was associated with CKD of at least mild stage. Similarly, there was a significant association between CKD of at least moderate stage and significant liver fibrosis (OR = 2.80, 95% CI = 1.27–6.18, *P* = 0.011) as measured by MRE (Table 4).

**Table 4:** Association of CKD with liver fibrosis in NAFLD.

Variables		≥ Mild						≥ Moderate					
		Univariable			Multivariable*			Univariable			Multivariable*		
		OR	95% CI	P-value	OR	95% CI	P-value	OR	95% CI	P-value	OR	95% CI	P-value
Age, years		1.03	1.02–1.04	<0.001	1.03	1.02–1.04	<0.001	1.03	1.01–1.05	0.005	1.02	1.0–1.04	0.072
Sex, Ref: female		0.75	0.58–0.98	0.032	0.90	0.68–1.20	0.478	0.73	0.41–1.30	0.284	0.93	0.48–1.77	0.812
Metabolic abnormalities													
	Central obesity	1.46	1.22–1.76	<0.001	1.34	1.10–1.63	0.004	3.26	1.98–5.38	<0.001	2.77	1.62–4.73	<0.001
	Hypertension	1.73	1.44–2.08	<0.001	1.33	1.09–1.62	0.005	2.69	1.75–4.14	<0.001	1.90	1.20–3.01	0.006
	Pre-diabetes	1.87	1.55–2.26	<0.001	1.48	1.22–1.81	<0.001	2.72	1.73–4.28	<0.001	1.95	1.20–3.17	0.008
	TG, ≥150mg/dL	1.24	1.03–1.48	0.025	1.24	1.02–1.51	0.029	1.78	1.16–2.74	0.009	1.52	0.96–2.39	0.075
	Decreased HDL-C	1.06	0.86–1.30	0.59	0.94	0.76–1.18	0.599	1.55	1.0–2.41	0.052	1.28	0.81–2.05	0.294
MRE categories (ref: F0)													
	F1	0.93	0.74–1.16	<0.001	0.90	0.72–1.14	0.383	1.29	0.72–2.32	0.040	1.32	0.72–2.45	0.370
	F2	1.51	1.03–2.21	0.670	1.19	0.80–1.77	0.388	3.84	1.80–8.16	0.011	2.80	1.27–6.18	0.011
	F3	2.90	1.66–5.05	0.001	2.0	1.13–3.55	0.018	2.95	0.83–10.53	0.365	1.70	0.46–6.30	0.425

*Adjusted for age, sex and metabolic abnormalities.

## Discussion

This study found that the prevalence of CKD was 12.4% in individuals with NAFLD who participated in health checkups. NAFLD was independently associated with CKD. However, when we separately applied a multivariable logistic regression analysis to the group of subjects with CKD of at least moderate stage, the association between NAFLD and CKD was attenuated after adjusting for age, and metabolically unhealthy status. In addition, CKD was significantly associated with the severity of liver fibrosis as measured by MRE in NAFLD.

Several cross-sectional studies [25–27] found that the prevalence of CKD ranged from 20% to 55% among patients with NAFLD, compared with 5–30% among those without NAFLD. Most studies have found that the association between NAFLD and increased prevalence of CKD persisted even after adjusting for CKD risk factors [3,15]. Consistent with previous studies, the present study found that the prevalence of CKD was higher among individuals with NAFLD, while it was lower than those found in hospital-based studies, which was attributed to the present study analyzing a community-based cohort.

In the present study, CKD was associated with the presence of NAFLD even after adjusting for age and a metabolically unhealthy status, defined as having two or more components of MS and/or diabetes. This confirmed the results of previous studies that the presence of NAFLD is strongly associated with increase in the prevalence and incidence of CKD [9]. However, when we separately applied a multivariable logistic regression analysis to the group of subjects with CKD of at least moderate stage, the association between NAFLD and CKD was attenuated after adjusting for age, and metabolically unhealthy status. Zhang et al. [28] analyzed the association between NAFLD and CKD using two population-based data sets from the US and China. Their subgroup analyses that divided subjects into the early and late stages of CKD revealed that NAFLD was associated with the early stages of CKD but not its late stages in both populations. Several studies have proposed that NAFLD impacts the development of CKD [29,30]. Possible pathophysiological mechanisms underlying how NAFLD contributes to the development and progression of CKD have been proposed [31,32]. NAFLD promotes hepatic insulin resistance and atherogenic dyslipidemia, induces hypertension, and causes the release of multiple proinflammatory cytokines that may contribute to the development and progression of CKD. However, the results of our study suggest that the presence of NAFLD impacts the development of the early stage of renal injury, whereas the synergistic effects of aging and metabolic abnormalities might be needed for the progression of later stages of CKD.

There is accumulating evidences that nonalcoholic steatohepatitis (NASH) is associated with CKD [3,9]. Histological resolution of NASH led to an improvement in renal function irrespective of weight loss [33]. Furthermore, a cross-sectional population-based study found that the incidence of all-cause, CVD-related, cancer-related, and other residual causes of mortality increased with the severity of CKD [34]. In the present study, CKD was significantly associated with severity of liver fibrosis as measured by MRE in NAFLD after adjusting for metabolic abnormalities. Moreover, the liver fibrosis scores (i.e., LS, NFS, and FIB-4, as estimations of hepatic fibrosis) were also higher in those with mild or moderate CKD than in those without CKD in the present study. The kidney biopsy-based study [35] showed that the liver fibrosis markers FIB-4 and NFS were negatively correlated with the eGFR in nephrosclerosis and IgA nephropathy. This result suggests that it is important to identify individuals with NAFLD early in the course of their disease and provide appropriate treatment and care to prevent negative outcomes.

Type 2 diabetes mellitus is the most common cause of CKD (diabetic nephropathy). Hypertension, glomerulonephritis, lupus, and inherited kidney disease also cause CKD. It is well known that the presence of comorbidities such as MS and diabetes with NAFLD increases the risk of CKD. However, there are insufficient data on the effect of each metabolic components of MS on the progression of NAFLD. In addition, the optimal threshold for the number of metabolic components of MS indicating that CKD screening is necessary not clear. We found that the presence of two or more metabolic components of MS and/or diabetes was an independent risk factors for CKD, regardless of the presence of NAFLD. In particular, in individuals with CKD of at least moderate stage, the presence of two or more metabolic components of MS and/or diabetes remained independent risk factors for CKD despite the effects of NAFLD being attenuated in the multivariable logistic regression analysis. Therefore, active screening for CKD is necessary when people have two or more metabolic components of MS and/or diabetes regardless of the presence of NAFLD.

This study has some limitations. First, selection bias could have been present due to the different reasons for seeking health checkups, such as MRE and abdominal US since MRE was performed only on those willing to pay for this additional test. In addition, males predominated in this study, which might have also caused selection bias and resulted in the study population not being representative of the broader Korean population. Second, fatty liver was only assessed using US. Although US can detect fat deposition in the liver, it is a subjective method for diagnosing fatty liver and cannot assess the disease severity. Third, the KDIGO definition indicates that CKD should be diagnosed over a period of >3 months. However, we could not follow the duration of abnormalities of kidney function due to the cross-sectional study design. Fourth, the prevalence of advanced fibrosis in this study was very low (only 1.6% of the subjects with NAFLD) due to the inclusion of community-based subjects. Fifth, we could not assess the type of antidiabetic and anti-hypertensive drugs in this study subjects. Previous reports apparently showed that the effect of antidiabetic drug such as sodium-glucose cotransporter 2 inhibitors (SGLT2i) on renal function and NAFLD. Effects of SGLT2 inhibition on blood pressure, sympathetic nerve activity, and inflammation could improve renal function in CKD [36,37]. And the cross-sectional design also means that further research is needed to determine causal relationships.

## Conclusions

Considering the burden imposed by the co-existence of NAFLD and CKD, it is necessary to identify individuals with NAFLD at a high risk of CKD in order to prevent the development and progression of CKD. Individuals with risk factors for CKD, such as being older and metabolically unhealthy and having significant or advanced liver fibrosis, should be provided with screening for CKD and appropriate treatment in order to delay or reverse the disease progression in NAFLD.

## Supporting information

S1 Data (XLSX)
